# Disentangling the Genetic Relationship Between Sleep and Epilepsy

**DOI:** 10.1002/acn3.70568

**Published:** 2026-11-02

**Authors:** Khang N. Le, Victoria E. Jackson, Rachel E. Stirling, Zachary F. Gerring, Remi Stevelink, Samuel F. Berkovic, Ingrid E. Scheffer, Philippa J. Karoly, Melanie Bahlo, Karen L. Oliver

**Affiliations:** ^1^ The Walter and Eliza Hall Institute of Medical Research Parkville Victoria Australia; ^2^ Department of Medical Biology The University of Melbourne Parkville Victoria Australia; ^3^ Department of Biomedical Engineering The University of Melbourne Parkville Victoria Australia; ^4^ Graeme Clark Institute of Biomedical Engineering The University of Melbourne Parkville Victoria Australia; ^5^ Department of Child Neurology, UMC Utrecht Brain Center University Medical Center Utrecht, European Reference Network EpiCARE Utrecht the Netherlands; ^6^ Department of Medicine, Epilepsy Research Center The University of Melbourne, Austin Health Heidelberg Victoria Australia; ^7^ Bladin‐Berkovic Comprehensive Epilepsy Program, Department of Neurology Austin Health Heidelberg Victoria Australia; ^8^ The Florey Institute and Murdoch Children's Research Institute Parkville Victoria Australia

**Keywords:** epilepsy, genetic overlap, shared loci, shared mechanisms, sleep

## Abstract

**Objective:**

A bidirectional relationship between sleep and epilepsy is well recognised, yet the biological mechanisms linking them remain poorly understood. Both have a genetic basis, with genome‐wide association studies (GWAS) capturing polygenic contributions. By leveraging GWAS summary statistics, we aimed to investigate their shared genetic architecture.

**Methods:**

We analysed seven sleep traits (morningness chronotype, sleep duration, habitual short/long sleep, insomnia, daytime napping/sleepiness) with common epilepsy types (focal epilepsy and genetic generalised epilepsy [GGE]). For each trait pair, we estimated genome‐wide genetic correlation, tested bidirectional causality, quantified polygenic overlap, performed gene‐trait associations to identify putative pleiotropic genes, estimated local genetic correlations and investigated whether regions of significance were driven by shared causal variants using colocalisation and fine‐mapping.

**Results:**

Genome‐wide analyses supported a positive genetic correlation between higher GGE risk and habitual short sleep, with extensive polygenic overlap (75%–97%) across seven sleep traits. Gene‐trait analyses identified *RMI1* as a putative sleep‐epilepsy candidate, with 13 additional sleep‐associated genes linked to monogenic epilepsies (e.g., *KANSL1*, *GRIN2A*). Locus‐specific analyses identified 16 genomic loci of interest (13 sleep‐GGE, 3 sleep‐focal epilepsy). Morningness‐GGE shared the largest number of locus‐specific associations. No significant causal relationships were identified, and causal variants did not colocalise.

**Interpretation:**

Our findings support complex shared genetic contributions between sleep and epilepsy, with distinct patterns for focal compared to generalised epilepsy. These differences may help explain syndrome‐specific sleep–wake associations, including seizure timing and predisposition. By localising shared loci and highlighting specific overlapping genes, we provide a foundation for future research into the mechanisms underlying epilepsy‐sleep comorbidities.

## 1 Introduction

Epilepsy is characterised by recurrent and unprovoked seizures [1]. It is a common, complex neurological disease with a wide array of aetiologies including genetic, structural and environmental factors [1, 2, 3].

It is well‐established that a bidirectional relationship between sleep and epilepsy exists. Epileptiform activity during sleep disturbs the quality of sleep, with nocturnal seizures leading to fragmented sleep, reduced sleep efficiency and altered sleep staging [4]. Notably, people with epilepsy commonly report poor sleep quality, excessive daytime sleepiness, and higher rates of sleep disorders compared to the general population [5, 6]. In the reverse direction, disruptions to sleep timing and duration can increase epileptiform activity and trigger seizures [7, 8]. Furthermore, certain epilepsy types and syndromes have distinctive seizure patterns associated with sleep–wake cycles. For instance, autosomal dominant sleep‐related hypermotor epilepsy—a focal epilepsy syndrome—presents with seizures occurring predominantly during sleep [9, 10]. In the genetic generalised epilepsies (GGEs), epilepsy with generalised tonic–clonic seizures alone and juvenile myoclonic epilepsy are often associated with seizures occurring shortly after awakening [11]. These clinical patterns, together with shared genetic determinants, such as circadian clock genes *RORA*, *RORB* and *CSNK1E* which have also been implicated in epilepsy [12, 13, 14, 15], further support an underlying relationship between epilepsy and sleep regulation.

Despite this widely recognised sleep‐epilepsy relationship, the biology underlying this association remains poorly understood. Previous observational studies exploring whether sleep timing and duration have a causal effect on seizure occurrence, have reported conflicting findings [7, 8, 16, 17, 18, 19]. These inconsistencies may be attributed to differences in study design, including varying sample sizes, heterogeneous patient populations and the use of a variety of different methods and devices to record seizures (e.g., self‐reported diaries, intracranial electroencephalography [EEG]) and sleep (e.g., EEG, smartwatches or self‐reported). Therefore, alternative analytical approaches that minimise these confounders, while scaling to larger populations, are needed.

The contribution of common variants to the polygenic architecture of sleep and epilepsy has been independently investigated by large‐scale genome‐wide association studies (GWAS) [20, 21, 22]. Analysing these genetic data together provides the opportunity to reveal biological links between sleep and epilepsy that traditional observational methods are unable to uncover. We aimed to reveal shared genetic associations and to identify specific candidate genes contributing to the complex relationship between sleep and epilepsy.

## 2 Materials and Methods

### 2.1 GWAS Summary Statistics

GWAS summary statistics for two common types of epilepsy and seven sleep‐related traits were retrieved for cross‐trait genetic analyses (Table 1). For epilepsy, this included focal epilepsy and GGE from the most recent International League Against Epilepsy (ILAE) GWAS [20]. For sleep traits, this included self‐reported ‘morningness’ chronotype (tendency to waking up early in the morning), sleep duration (sleep hours/day), habitual short sleep (sleep < 7 h/day), habitual long sleep (sleep > 9 h/day), daytime napping, daytime sleepiness and insomnia [21, 22, 23, 24, 25]. All summary statistics were derived from European ancestry populations. Seven sleep traits are self‐reported measures, rather than monitor‐derived (e.g., accelerometer, wearables) measures, selected for their substantially larger sample sizes within UK Biobank and public availability.

**TABLE 1:** Sample sizes in GWAS summary statistics for epilepsy types and sleep traits. Sample sizes for cases and controls are reported for binary traits. Total sample size alone is reported for quantitative and ordinal traits.

Group	Trait	Population	Sample size (*N*)			Sample source	GWAS source
			Case	Control	Total		
Epilepsy	Focal epilepsy [20]	European	14,939	42,436	57,375	ILAE Consortium	epiGAD
	Genetic generalised epilepsy (GGE) [20]	European	6952	42,436	49,388	ILAE Consortium	epiGAD
Sleep‐related	Sleep duration [21]	European	—	—	446,118	UK Biobank	GWAS Catalog (GCST007561)
	Habitual long sleep (sleep > 9 per 24 h) [21]	European	34,184	305,742	339,926	UK Biobank	GWAS Catalog (GCST007560)
	Habitual short sleep (sleep < 7 per 24 h) [21]	European	106,192	305,742	411,934	UK Biobank	GWAS Catalog (GCST007559)
	‘Morningness’ chronotype [22]	European	252,287	150,908	403,195	UK Biobank	GWAS Catalog (GCST007565)
	Insomnia [23]	European	109,402	277,131	386,533	UK Biobank	CNCR Complex Trait Genetics lab
	Daytime napping [24]	European	—	—	452,633	UK Biobank	GWAS Catalog (GCST011494)
	Daytime sleepiness [25]	European	—	—	452,071	UK Biobank	Sleep Disorder Knowledge Portal

*Note:* GWAS Catalog (https://www.ebi.ac.uk/gwas/); CNCR: Center for Neurogenomics and Cognitive Research (https://cncr.nl/research/summary_statistics/); Sleep Disorder Knowledge Portal (https://sleep.hugeamp.org/datasets.html); epiGAD: The Epilepsy Genetic Association Database (https://www.epigad.org/download/final_sumstats.zip).

Abbreviations: GWAS, genome‐wide association study; ILAE, International League Against Epilepsy.

GWAS summary statistics were utilised across two complementary analytical streams: global analyses and local analyses, designed to address a series of research questions (Figure 1).

**FIGURE 1:**
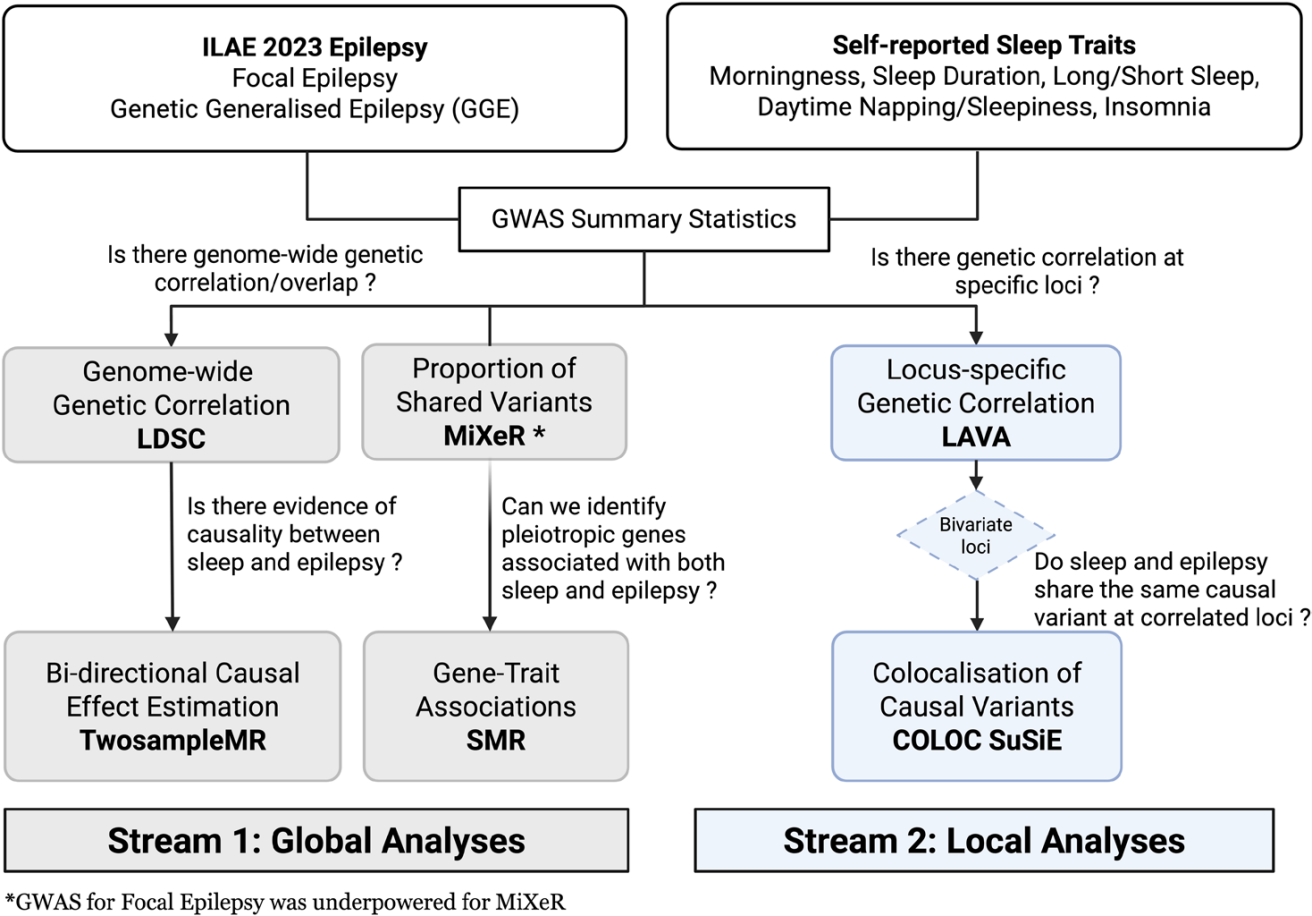
Analytical framework for investigating genetic relationships between sleep and epilepsy. GWAS summary statistics from the ILAE 2023 epilepsy study (genetic generalised epilepsy, focal epilepsy) and seven self‐reported sleep traits from multiple studies (Table 1) were analysed using two complementary streams. Stream 1 (global analyses) assessed genome‐wide genetic correlations with Linkage Disequilibrium Score Regression (LDSC). Bidirectional Mendelian Randomisation was used to evaluate whether sleep‐epilepsy trait pairs with significant LDSC correlation show evidence of a causal relationship. MiXeR analyses were also undertaken to estimate the overlap of causal variants contributing to each trait pair. Of note, GWAS for focal epilepsy was underpowered for MiXeR. Additionally, summary‐data‐based Mendelian Randomisation (SMR) was used to prioritise genes underlying GWAS signals by integrating brain tissue eQTL data, towards identifying genes jointly associated with sleep traits and epilepsy. Stream 2 (local analyses) used Local Analysis of [co]Variant Association (LAVA) at 2495 genomic loci to identify regions with significant localised genetic correlation. For loci showing significant LAVA correlations, colocalisation (COLOC SuSiE) was applied to determine whether sleep and epilepsy are predicted to share the same causal genetic variants at the locus. Detailed code for analyses and plotting was made publicly available through the Github repository: https://github.com/bahlolab/SleepEpi_postgwas_manuscript/. This figure was created with BioRender.com.

### 2.2 Genome‐Wide Genetic Correlation

Linkage Disequilibrium Score regression (LDSC) [26, 27] was used to estimate genome‐wide genetic correlation between each sleep and epilepsy trait pair across the entire genome. To ensure robust genetic signal, we calculated the heritability *Z*‐statistic (SNP‐based heritability/standard error) for each trait to confirm that the proportion of the trait variance explained by GWAS SNPs was significantly different from zero (i.e., *Z*‐statistic > 2) (Supplementary Table 1, Supplementary Figure 1). For binary traits, SNP‐based heritability was converted to a liability scale using population prevalence. To control for multiple testing in genetic correlation analyses, we applied Bonferroni multiple testing correction (*p* < 0.05/14 sleep‐epilepsy trait pairs).

### 2.3 Bidirectional Causal Effect Estimation

To investigate potential causal relationships between sleep and epilepsy, we employed Mendelian randomisation (MR) as implemented in the TwosampleMR R package [28]. In forward analyses, we estimated the causal effect of each sleep‐related trait (exposure) on each epilepsy trait (outcome). In reverse analyses, we treated epilepsy as the exposure and sleep traits as the outcome (for further details see Supplementary Notes 1 and 2).

### 2.4 Proportion of Shared Variants

A bivariate causal mixture model (MiXeR) [29] was used to estimate the polygenic overlap between sleep traits and epilepsy. Rather than identifying the specific genomic location of causal variants, MiXeR fits a likelihood model to the observed pattern of GWAS summary statistics. This model quantifies the number of variants that affect each trait separately, and the number that affect both traits, providing an estimate of shared causal variants irrespective of effect direction (e.g., concordant or discordant) [30]. Note that the focal epilepsy GWAS was underpowered for this analysis, hence not reported.

### 2.5 Gene‐Trait Associations

To identify pleiotropic genes influencing both sleep traits and epilepsy, we employed summary‐data‐based Mendelian randomisation (SMR). This approach integrates GWAS summary statistics with brain‐specific expression quantitative trait loci (eQTLs) from BrainMeta, derived from 2865 human postmortem brain cortex tissue samples of European ancestry [31]. Using the online SMR portal with default parameters [32, 33], we determined significant gene‐trait associations using Bonferroni correction (*p* < 0.05/10,534 tested genes). This was done separately for each trait. Significant SMR genes for the seven sleep traits were then intersected with those for epilepsy. We further cross‐referenced the SMR genes with 22 known circadian clock genes [34, 35, 36, 37] (Supplementary Table 2) and a curated resource of monogenic epilepsy genes, Genes4Epilepsy (1078 genes; updated March 2026) [3]. For simplicity, these gene sets will now be referred to as ‘sleep‐associated’ and ‘epilepsy‐associated’ genes.

### 2.6 Locus‐Specific Genetic Correlation

We performed Local Analysis of [co]Variant Association (LAVA) [38] to identify specific genomic regions significantly correlated with both sleep and epilepsy traits. Specifically, we analysed 2495 pre‐partitioned loci provided with the LAVA software. For each trait, we scanned across all loci to identify those with significant univariate associations using Bonferroni correction (*p* < 0.05/2495). Loci that were significantly associated with both a sleep and epilepsy trait by univariate analyses were then tested for local pairwise genetic correlation. Significant bivariate loci were determined according to the Benjamini–Hochberg procedure.

We matched the LAVA‐defined loci (chromosome:start–stop) to their corresponding cytogenetic location, for example chr3:47588462–50387742 was mapped to 3p21.31. The conversion step was completed using the GenomicRanges R package and UCSC Genome Browser human hg19 reference. We then cross‐referenced each significant region with epilepsy‐associated genes from the Genes4Epilepsy repository [3] and genes important in sleep regulation (Supplementary Table 2) [34, 35, 36, 37].

### 2.7 Colocalisation of Causal Variants

To investigate whether shared regions identified by LAVA are driven by the same or different causal variants, we performed colocalisation using COLOC with default priors [39]. COLOC calculated posterior probabilities (PP) for whether the lead SNP at each significant LAVA bivariate locus was independent or shared by the sleep‐epilepsy trait pair. For loci with COLOC‐PP.H4 ≥ 0.8, indicating strong evidence for a shared causal variant, we applied the Sum of Single Effects (SuSiE) [40] to fine‐map the region and identify the most likely causal variant (see Supplementary Notes 1 and 2). This two‐step approach is consistent with current practice in genetic colocalisation studies to account for multiple causal variants within complex linkage disequilibrium [40].

## 3 Results

### 3.1 Genome‐Wide Genetic Correlation

Using LDSC to test genome‐wide genetic correlations (global *r*_g_) between sleep traits and epilepsy, we identified two significant associations (*p*_adj_ ≤ 0.05, Figure 2, Supplementary Table 3). Short sleep was positively correlated with GGE (global *r*_g_ = 0.16, *p*_adj_ = 0.003), while sleep duration showed a negative correlation with GGE (global *r*_g_ = −0.12, *p*_adj_ = 0.03). Notably, these signals reflect the same underlying relationship. Sleep duration is a continuous trait (self‐reported overall sleep duration hours/day), whereas short sleep is a binary trait (sleep duration < 7 h/day) derived from the same overall sleep duration data [21].

**FIGURE 2:**
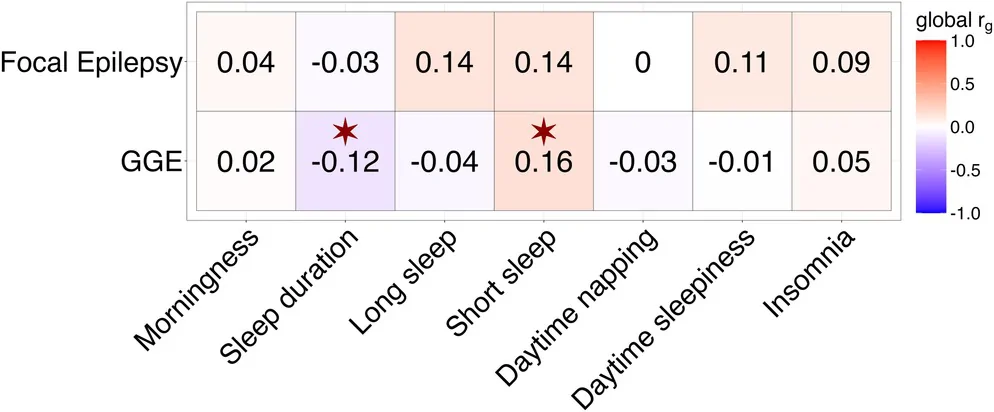
LDSC results showing genome‐wide genetic correlation (global *r*_g_) between sleep‐related traits and epilepsy types. Genetic correlation values are indicated by colour, with red representing positive correlations and blue representing negative correlations. Colour intensity reflects the magnitude of global *r*_g_ as shown in the scale bar, with white indicating correlations near or at zero. Numerical values within each cell show the global *r*_g_ estimate. Statistical significance was determined using Bonferroni correction for multiple testing, with ✶ denoting significant correlations (*p* ≤ 0.05/14 tests).

### 3.2 Bidirectional Causal Effect Estimation

While genome‐wide genetic correlation analyses revealed shared genetic influences between sleep traits and epilepsy, they do not address whether the nature of this relationship could be causal. To test whether short sleep and sleep duration causally influence GGE risk or vice versa, we performed bidirectional two‐sample Mendelian randomisation (MR). We used the independent genome‐wide significant SNPs for sleep traits and epilepsy as instrumental variables (Supplementary Table 4).

Applying our pre‐specified criteria for significant causal inference (Supplementary Notes 1 and 2), no sleep‐epilepsy relationships were established as causal in either the forward or reverse direction (Supplementary Table 5).

### 3.3 Proportion of Shared Variants

Genome‐wide genetic correlation analyses assume concordant SNP effect direction. We next sought to estimate shared genetic effects, while accounting for mixed‐directional SNPs, by performing MiXeR analyses. MiXeR results for sleep traits were consistent with high polygenicity, where each trait was estimated to have 6.5k–9.3k trait‐influencing variants. This was approximately three times higher than the estimate for GGE (2.94k variants) (Supplementary Table 6).

Bivariate MiXeR analyses revealed extensive cross‐trait genetic overlap: 97% (2.86k/2.94k) and 94% (2.76k/2.94k) of GGE predicted causal variants were shared with sleep duration and short sleep, respectively (Supplementary Figure 2). Furthermore, MiXeR estimated GGE to also share 75%–85% of causal variants with the remaining five sleep traits. Unlike LDSC, MiXeR does not provide a formal significance test for these overlap estimates; however, each estimate reflects the mean of 20 independent model fits (±SD; Supplementary Table 7), which showed only small variation, indicating the estimates are numerically stable. Notably, these five sleep traits are not significantly correlated with GGE, thus this result likely reflects shared variants with opposing effect directions, which cancel each other out in a global genetic correlation analysis.

### 3.4 Gene‐Trait Associations

From our SMR analysis using human brain tissue eQTL data, we identified 115 genes significantly associated with sleep traits and six genes associated with GGE, after correcting for 10,534 tested genes (Supplementary Table 8). No genes met our stringent statistical threshold for focal epilepsy.

One gene demonstrated pleiotropic effects on both sleep and epilepsy: *RMI1* was significantly associated with GGE (*β* = 0.22, *p*_adj_ = 6.93 × 10^−4^) and daytime napping (*β* = 0.03, *p*_adj_ = 0.009) (Figure 3). Fifteen significant SMR hits also overlapped with epilepsy‐associated genes from the Genes4Epilepsy repository, including two epilepsy SMR hits (*AP3D1*, *HNRNPK*) and 13 sleep SMR hits.

**FIGURE 3:**
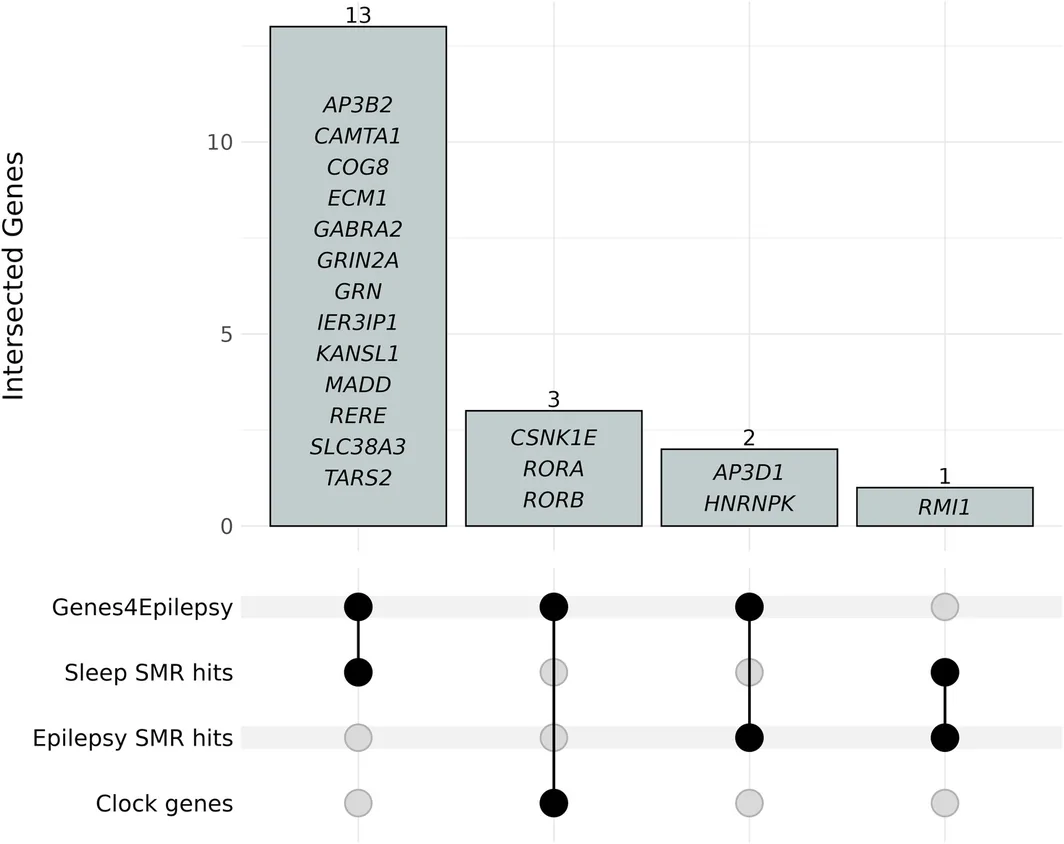
Overlap between SMR genes, circadian clock genes and known epilepsy genes. Upset plot showing the overlap of genes identified in four gene sets: significant SMR gene‐trait associations for sleep traits (Sleep SMR hits) and epilepsy (Epilepsy SMR hits), the Genes4Epilepsy repository (1078 genes; March 2026 release) [3] and 22 known circadian clock genes [34, 35, 36, 37]. Each vertical bar represents a distinct subset of intersected genes from multiple gene sets as indicated by the black filled circles and connecting line. The height of each vertical bar represents the number of genes in that intersection, which are named inside. For example, the largest vertical bar represents 13 genes present in both Sleep SMR hits and the Genes4Epilepsy repository.

These overlapping genes, strongly associated with both sleep traits and known monogenic epilepsy genes, support shared genetic mechanisms underlying epilepsy pathogenesis and sleep‐associated traits.

Global analyses (Stream 1; Figure 1) revealed a large genetic overlap between GGE and all sleep traits, even those lacking significant genome‐wide genetic correlation, with MiXeR analyses indicating that mixed‐directional SNP effects contribute to this pattern. Direct comparison of genome‐wide significant lead SNPs identified only three GGE risk loci overlapping with genome‐wide significant loci for daytime napping, sleep duration, short sleep and morningness (Supplementary Table 9), indicating that most shared signal is not captured at the level of individual genome‐wide significant hits. To identify the specific genomic loci underlying these opposing correlation signals, we performed local genetic correlation analyses as part of our Stream 2 set of analyses.

### 3.5 Locus‐Specific Genetic Correlation

Scanning 2495 loci across the genome, we identified 20 significant bivariate correlations (*p*_adj_ ≤ 0.05) between sleep traits and epilepsy at 16 cytogenetic regions (Figure 4; Supplementary Tables 10 and 11). These included 2q32.2–q32.3, where GGE and morningness exhibit the strongest positive local genetic correlation (local *r*_g_ = 1, *p*_adj_ = 9.9 × 10^−6^), and 2p16.1, where GGE and sleep duration showed the strongest negative correlation (local *r*_g_ = −1, *p*_adj_ = 9.8 × 10^−6^).

**FIGURE 4:**
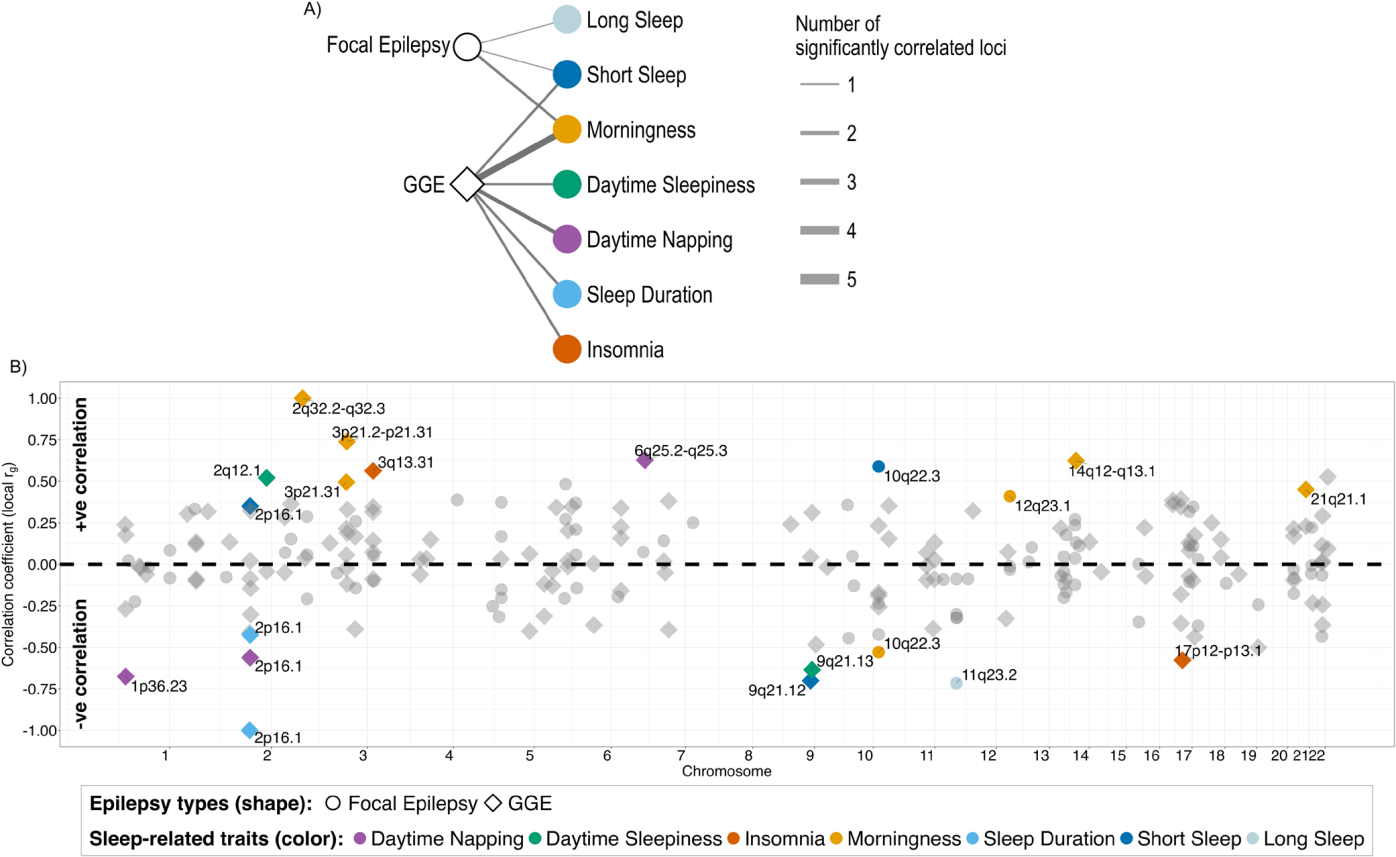
Local genetic correlations between epilepsy and sleep traits identified by LAVA. (A) Network diagram summarising the number of significant local genetic correlations (local *r*_g_) between each sleep‐epilepsy trait pair. Edge thickness indicates the number of loci with significant local *r*_g_ (*p*_adj_ ≤ 0.05) after Benjamini–Hochberg correction. (B) Miami plot displaying locus‐specific genetic correlations across all chromosomes. Each data point represents a bivariate correlation signal between a sleep‐epilepsy trait pair. Local *r*_g_ values range from −1 (negative) to 0 (no correlation) and +1 (positive). Data point shapes indicate epilepsy types: circles for focal epilepsy and diamonds for genetic generalised epilepsy (GGE). Data point colours denote sleep‐related traits according to the colour legend. Statistically significant local correlations (*p*_adj_ ≤ 0.05) are shown in colour, while nominally significant and non‐significant correlations appear in grey. Loci with significant local correlation are labelled with their corresponding cytogenetic regions.

Sleep‐epilepsy traits with significant local correlations were connected using a network diagram (Figure 4A). This revealed 16 connections for GGE across all sleep traits, except long sleep, at 13 distinct cytogenetic regions. GGE‐morningness had the highest number of significant correlations (*n* = 5), followed by GGE‐daytime napping (*n* = 3). Focal epilepsy had four connections with morningness, long sleep and short sleep, at three cytogenetic regions (Figure 4B). The significant focal epilepsy connections with sleep traits were all distinct from those with GGE.

Cross‐checking genes encompassed by the 16 significant cytogenetic regions against the Genes4Epilepsy resource identified 22 epilepsy‐associated genes, as well as two genes regulating sleep and circadian rhythm (*PER3, RORB*), detailed in Supplementary Table 12. Six genes were previously identified as significantly associated with sleep traits by the SMR analyses, including three established monogenic epilepsy genes (*CAMTA1*, *RERE*, *SLC38A3*) [3].

While *RMI1* was identified as a pleiotropic gene associated with sleep and epilepsy at the gene level, its encompassing locus did not survive our stringent multiple testing correction for LAVA's locus‐specific genetic correlation analyses.

### 3.6 Colocalisation of Causal Variants

COLOC was applied to the 16 significant cytogenetic regions identified by LAVA. These analyses identified four loci with COLOC‐PP.H4 ≥ 0.8 (Supplementary Table 13), providing suggestive evidence for shared sleep‐epilepsy causal variants within three cytogenetic regions (i.e., 2p16.1, 2q32.2‐q32.3 and 3p21.2‐p21.31). Since COLOC assumes a single causal variant per region, we applied SuSiE fine‐mapping to the four loci with COLOC‐PP.H4 ≥ 0.8. The SuSiE‐based colocalisation approach failed to conclusively confirm the presence of shared causal variants at these loci (Supplementary Table 14).

## 4 Discussion

A bidirectional relationship between sleep and epilepsy has long been recognised in clinical practice. Here, leveraging large‐scale GWAS datasets, we provide strong evidence for a genetic underpinning to this relationship. Specifically, we identified shared genetic architecture between epilepsy and multiple sleep‐related traits at genome‐wide, locus‐specific and single‐gene levels. This highlights both the complex polygenic overlap between sleep and epilepsy, and the value of applying multiple complementary genomic approaches to fully characterise cross‐trait associations.

Genome‐wide genetic correlation analyses using LDSC revealed GGE to be negatively correlated with sleep duration and, in turn, positively correlated with short sleep. These results suggest that a genetic predisposition to needing less sleep is linked to higher genetic risk for GGE and vice versa. Short sleep duration has been causally linked to depression and more broadly associated with other psychiatric and neurological conditions [41, 42], reflecting the growing recognition that sleep trait genetics may be important contributors to disease risk. Our subsequent two‐sample Mendelian randomisation analyses examining bidirectional causality found no evidence to support a direct causal relationship between sleep duration and GGE, in either direction. This suggests that the observed sleep‐epilepsy genetic correlations likely reflect a shared polygenic basis rather than direct causality.

Despite the strong clinical relationship between sleep and epilepsy, the genetic overlap has not been rigorously examined with a focus on different epilepsy types [43], nor with the systematic incorporation of multiple complementary cross‐trait analytical methods [41, 44]. This is important, not only because of the differences in genetic architecture between focal and generalised epilepsies [20], but because each analytical method has its own strengths and limitations. For example, genome‐wide genetic correlation analyses using LDSC provide only one collapsed view of shared genetic architecture, where variants with opposing effect directions can cancel out, masking the true extent of overlap.

We applied MiXeR to account for this, revealing a far broader pattern of genetic sharing than LDSC alone would suggest. Sleep traits were estimated to be three times more polygenic than GGE, and surprisingly, between 75% and 97% of GGE‐associated variants are shared across all seven sleep traits investigated. Among the shared variants, however, only 43%–55% act concordantly, meaning close to half act in opposing directions between GGE and each sleep trait. This near‐balanced mix of concordant and discordant shared variants offers a plausible explanation for why genome‐wide genetic correlation failed to detect broader pairwise sleep‐epilepsy relationships. Variants with mixed‐effect directions could also have contributed to the negative two‐sample Mendelian randomisation results, albeit on a smaller scale as these analyses were restricted to genome‐wide significant variants [30]. MiXeR's ability to quantify genetic overlap regardless of effect direction has proven valuable across complex trait research more broadly [30, 45]. Our MiXeR results suggest that the genetic relationship between sleep and epilepsy may extend beyond the association with the ‘need for less sleep’ captured by LDSC. We note that, due to linkage disequilibrium, MiXeR's estimate of shared causal variants may reflect nearby variants in linkage rather than a single variant with a true pleiotropic effect on both traits. Therefore, the estimated proportion of overlap between trait pairs should be interpreted with caution.

The compelling genetic overlap between GGE and all sleep traits motivated us to perform locus‐specific genetic correlation analyses to identify specific genomic regions associated with both sleep and epilepsy. These analyses revealed genetic associations between sleep‐epilepsy traits across different loci and comprised a mix of positive and negative effect directions that differed between focal epilepsy and GGE. The largest number of significant connections was found between GGE and the morningness chronotype. This is a particularly interesting observation. Individuals with GGE syndromes, juvenile myoclonic epilepsy and epilepsy with generalised tonic–clonic seizures alone have a predilection to seizures on awakening, which are further exacerbated by sleep deprivation. While these mixed‐effect results were expected from our MiXeR analyses, they importantly build on them by localising specific genomic regions that can be prioritised for functional investigation of the underlying sleep‐epilepsy biology.

Linking GWAS loci back to causal genes and variants is a challenging but important downstream step for all complex trait investigations. We sought to identify specific genes contributing to the underlying sleep‐epilepsy biology by leveraging both brain gene expression data and cross‐referencing known sleep‐associated and epilepsy‐associated genes. We used SMR to ask whether variation in a gene's expression is likely to be causally or pleiotropically associated with any sleep trait or epilepsy type. This analysis identified brain expression of *RMI1* as the only significant gene‐trait association for both sleep and epilepsy. Despite *RMI1*'s established role in genomic stability and DNA damage response [46], the mechanisms connecting it to sleep and epilepsy remain unclear. Future functional studies will be needed to determine whether this represents a true biological link or a statistical artefact of shared genomic architecture.

Overlapping the remaining genes identified by SMR for sleep or epilepsy with established sleep and monogenic epilepsy‐associated genes highlighted *GRIN2A* and *KANSL1* as potential candidates contributing to the underlying sleep‐epilepsy network. Brain expression of *GRIN2A*, a well‐established monogenic epilepsy gene, was significantly associated with sleep traits. *GRIN2A* is an important cause of developmental and/or epileptic encephalopathy with spike‐and‐wave activation in sleep (D/EE‐SWAS)—a severe epilepsy syndrome associated with spike‐and‐wave activation in sleep [47]. In rare cases, *GRIN2A* pathogenic variants have also been associated with self‐limited epilepsy with centro‐temporal spikes (SeLECTS), in which brief focal seizures typically occur during early sleep or 1–2 h prior to awakening [48]. Consistent with this, *Grin2a*‐deficient mice exhibit sleep‐related epileptiform discharges and transient alterations in slow‐wave sleep during development, providing independent support for a mechanistic link between *GRIN2A* and sleep‐epilepsy comorbidity [49]. In addition, *KANSL1* showed broad associations with multiple sleep traits in our SMR analyses, including daytime napping, daytime sleepiness, sleep duration and long sleep [50]. Pathogenic variants in *KANSL1* cause Koolen‐de Vries syndrome, in which up to half of the individuals develop epilepsy that shares features with SeLECTS, including seizures triggered by sleep [51].

The same gene cross‐referencing approach was taken for the local genomic regions identified by LAVA. This highlighted key candidate genes at 1p36.23 (*CAMTA1*, *RERE*) and 3p21.31 (*SLC38A3*). These are established monogenic epilepsy‐associated genes now associated with sleep traits, including long sleep, daytime napping and morningness chronotype. Notably, *CAMTA1* is located near the core circadian clock gene *PER3* at 1p36.23 (Supplementary Figure 3). Meanwhile *RORB*, another clock gene with established GGE and developmental and epileptic encephalopathy associations [13, 14], is located at the 9q21.13 locus associated with GGE‐daytime sleepiness. *RORB* was not tested for gene‐trait associations due to its absence from the brain eQTL dataset. Given *RORB'*s direct relevance to both GGE and circadian biology, this gap warrants targeted future investigation.

While the colocalisation and fine‐mapping results were not consistent with shared sleep‐epilepsy causal variants, this does not preclude different variants in the same genes and/or gene networks contributing to the observed sleep‐epilepsy relationship. It is also possible that current epilepsy GWAS, particularly for focal epilepsy, remain underpowered to detect shared causal variants, and that larger samples may yet reveal colocalising signals. That said, our current results support both focal and generalised epilepsy sharing genetic architecture with specific sleep traits at distinct genomic regions. The differing relationships between focal and generalised epilepsies with various sleep traits are unsurprising given their phenotypic and genetic heterogeneity and may help explain why seizure‐sleep associations vary across diagnostic classifications. Extending these analyses to specific epilepsy syndromes will be an important next step; while syndrome‐level GWAS remain underpowered [20], calculating sleep polygenic risk scores in large, stratified cohorts represents a complementary approach to identify whether specific sleep genetic profiles are enriched across distinct clinical presentations.

Our results should be interpreted with several limitations in mind. First, as mentioned above, current epilepsy GWAS sizes remain modest, which may have limited our ability to detect important biological signals. In an attempt to address this, we analysed broad epilepsy groups (GGE, focal epilepsy) rather than specific sleep‐related epilepsy syndromes, which would be even more underpowered with smaller sample sizes. Despite this, the broad focal epilepsy GWAS summary statistics remained underpowered for MiXeR analysis. Second, analyses were restricted to European‐ancestry individuals, limiting generalisability. Future studies incorporating more diverse ancestries are needed to determine whether these genetic relationships hold across populations. Third, we prioritised loci with significant local genetic correlation signals; while this focused analysis on the strongest regions, we may have missed colocalised variants at loci that were only nominally significant. Fourth, although our multi‐method approach was comprehensive, the lack of alignment across methods introduces some uncertainty in interpreting individual results; nonetheless, triangulating across methodologically distinct approaches provides a more robust basis for inference than any single method could offer alone. Fifth, the sleep traits included in our analyses were based on self‐report, whereas epilepsy cases were ascertained through neurologist referral and diagnosis; this asymmetry in phenotyping rigour means that misclassification or recall bias in the sleep measures may have attenuated or otherwise distorted the genetic relationships we identified. Future studies incorporating objectively measured sleep data, for example from wearable devices, would help address this recall bias and provide a valuable complement to self‐reported measures. Finally, other Mendelian randomisation studies on this topic have reported differing conclusions regarding causality [43, 44, 52], which may reflect their use of less well‐powered epilepsy GWAS summary statistics [44, 52], or their analysis of ‘all epilepsy’ as a single phenotype rather than separating the genetically and clinically distinct GGE and focal subtypes, as we did here [43, 44].

Overall, we identified extensive genetic overlap between the common epilepsies and seven sleep‐related traits. We localised contributing genomic regions and highlighted specific candidate genes underlying these associations. These findings advance our biological understanding of the sleep‐epilepsy relationship and raise the question of whether sleep‐targeted clinical interventions may represent a viable strategy for improving seizure control.

## Author Contributions

K.L.O. and M.B. conceived and supervised the study. K.N.L. performed all analyses. K.N.L. and K.L.O. drafted the manuscript. All authors provided critical intellectual input and approved the final manuscript.

## Funding

K.L.O., Z.F.G. and M.B. are supported by Investigator Grants from the National Health and Medical Research Council (APP2040880, APP2034743 and APP1195236). I.E.S was supported by the National Health and Medical Research Council (GNT1091593, GNT1172897, GNT2006841, GNT2010562, GNT2033247) and Medical Research Future Fund (GNT2007707).

## Ethics Statement

The authors have nothing to report.

## Consent

The authors have nothing to report.

## Conflicts of Interest

The authors declare no conflicts of interest.

## Supporting information


**Supplementary Figure 1:** Top forest plot shows SNP‐based heritability (*h* ^2^_SNP_) estimates for two common epilepsies and seven sleep‐related traits. For each trait, *h* ^2^_SNP_ estimate with 95% confidence intervals are shown. Bottom forest plot shows *Z*‐statistic (LDSC *h* ^2^_SNP_/SE). Red vertical dashed line indicates cut‐off where only GWAS with *Z*‐statistic > 2 were included in post‐GWAS analysis. Note that *h* ^2^_SNP_ estimates reported in the ILAE Consortium on Complex Epilepsies 2023 GWAS manuscript were derived using LDAK resulting in overall higher *h* ^2^_SNP_ estimates than those reported here.
**Supplementary Figure 2:** Proportion of shared variants between genetic generalised epilepsy (GGE) and sleep traits, estimated by MiXeR. Venn diagrams show the overlap between GGE‐influencing variants (blue circles) and variants associated with each sleep trait (orange circles). The overlapping region indicates the number of variants shared between GGE and each sleep trait, and the proportion of these shared variants with concordant versus discordant directional effects, as labelled within each panel. Note that GWAS summary statistic for focal epilepsy was underpower for MiXeR, hence not reported.
**Supplementary Figure 3:** UCSC visualisation of genes locate in 1p36.23 (chr1:7711795–8580987; hg 19 reference).
**Supplementary Note 1:** Detailed procedure for bidirectional Mendelian randomisation.
**Supplementary Note 2:** Detailed procedure for colocalisation of causal variants.

**Supplementary Table 1** GWAS information and LDSC SNP‐based heritability.
**Supplementary Table 2:** Collection of clock genes.
**Supplementary Table 3:** LDSC‐global correlation results.
**Supplementary Table 4:** Instrumental variant (SNP) used in bivariate two‐sample Mendelian randomisation.
**Supplementary Table 5:** Bidirectional two‐sample Mendelian randomisation results.
**Supplementary Table 6:** Univariate MiXeR analysis.
**Supplementary Table 7:** Bivariate MiXeR analysis.
**Supplementary Table 8:** SMR gene‐trait associations.
**Supplementary Table 9:** Overlapping genome‐wide significant risk loci between GGE and sleep‐related traits.
**Supplementary Table 10:** Univariate LAVA analysis results.
**Supplementary Table 11:** Bivariate LAVA—local correlation analysis results.
**Supplementary Table 12:** Overlap of genes in 16 LAVA significant cytogenic regions with Genes4Epilepsy and known clock genes.
**Supplementary Table 13:** COLOC‐based colocalisation analysis results.
**Supplementary Table 14:** SuSiE‐based colocalisation analysis results.

## Data Availability

This project was conducted using publicly available GWAS summary statistics and statistical tools, which can be found from the references.
