# Supplementary material for: Disentangling the Genetic Relationship Between Sleep and Epilepsy
Source: Ann Clin Transl Neurol. 2026 Nov 2:10.1002/acn3.70568. Online ahead of print. doi: 10.1002/acn3.70568 (PMC13728479; doi:10.1002/acn3.70568)
Supplement: Supplementary file 1 [file ACN3-9999-0-s002.docx]

**Disentangling the Genetic Relationship Between Sleep and Epilepsy**

**Supplementary Notes**

**Supplementary Note 1: Detailed procedure for bi-directional Mendelian Randomisation**

**Supplementary Note 1 Figure: Schematic illustration of Mendelian Randomisation framework.** This approach uses genetic variants (SNPs) as instrumental variables to estimate causal effect of an exposure trait on an outcome trait. For valid causal inference, SNP instruments of an exposure trait must satisfy three core assumptions: Assumption 1: selected SNPs must be strongly associated with the exposure traits; Assumption 2: selected SNPs must not be associated with confounding factors that influence either the exposure or outcome; and Assumption 3: selected SNPs must affect the outcome exclusively through their effect on the exposure trait. In this study, we applied bidirectional MR by interchanging the role of sleep and epilepsy traits to assess potential causation from both directions. Created with BioRender.com

For TwosampleMR analyses, SNP instruments are used as genetic proxies for the exposure trait(s) which permits inference of causality rather than mere association/correlation. To select suitable SNP instruments satisfying the three core assumptions of MR framework, we used FUMA (<https://fuma.ctglab.nl/>) [1], a web-based tool with default settings to identify independent lead SNPs for each exposure trait. When specific SNP instruments were absent from the outcome summary statistics, we identified proxy SNPs using the LDproxy function from the LDlinkR package [2], applying LD r^2^ ≥0.8 in EUR populations. To ensure robust instrumental strength for causal effect estimation, only exposure traits with more than five SNP instruments with F-statistic ≥10 was included for Twosample MR analyes.

For causal effect estimation, the Inverse Variance Weighted (IVW) method was the primary analytical approach. To ensure the robustness and validity of significant IVW findings, sensitivity analyses were conducted using alternative MR methods operating under different assumptions. These included MR-Egger regression and Weighted Median. Bonferroni procedure was applied for multiple testing correction. To evaluate potential horizontal pleiotropy, we examined the MR-Egger intercept using the *mr_pleiotropy_test* function, where a p-value ≤0.05 indicated evidence of directional pleiotropy. Additionally, we evaluated heterogeneity among SNP instruments using Cochran’s Q test with *mr_heterogeneity* function, where p-value ≤0.05 suggested significant heterogeneity among SNP instruments. Finally, we generated leave-one-out plots using the *mr_leaveoneout_plot* function to identify influential outliers that might bias causal effect estimation.

Causal inferences that survived Bonferroni correction (p <0.05/6 tests per direction) for IVW primary analyses and passed sensitivity analyses (MR-Egger regression, Weighted Median, MR-Egger intercept and Q-test) are deemed significant.

**Supplementary Note 2: Detailed procedure for Colocalisation of Causal Variants**

**Supplementary Note 2 Figure: COLOC colocalisation framework distinguishing shared versus distinct causal variants**. Schematic illustration of two scenarios tested by Bayesian colocalisation framework. Left panel: A single causal SNP influence both epilepsy and sleep-related traits, representing true genetic pleiotropy (i.e., shared causal variants; COLOC PP.H4 ≥0.8). Right panel: Two distinct causal SNPs within the same linkage disequilibrium block independently influence epilepsy and sleep-related traits, respectively (evidence for distinct causal variants; PP.H3 ≥0.8). While both scenarios can produce genetic correlation between traits, the left scenario indicates a shared causal variant. Created with BioRender.com

COLOC operates on Bayesian framework to evaluate five hypotheses **H0:** No association with either trait, **H1:** Association with trait 1, **H2:** Association with trait 2, **H3:** Association with both trait 1 and trait 2 via two independent causal variants, **H4:** Association with both trait 1 and trait 2 via a shared causal variant. We considered PP.H4 ≥0.8 as strong evidence for a shared causal SNP indicating genetic colocalisation of the trait pair, and PP.H3 ≥0.8 as strong evidence that the locus is associated with both traits but via different causal variants.

Of note, COLOC assumes a single causal variant per region. To account for the possibility of multiple independent association signals within these regions, we next applied Sum of Single Effects (SuSiE) [3] fine-mapping to define credible sets of causal variants for each trait, followed by colocalisation testing between each pair of credible sets.

**Supplementary Figures**

**Supplementary Figure 1:** Top forest plot shows SNP-based heritability (h^2^_SNP;_) estimates for two common epilepsies and seven sleep-related traits. For each trait, h^2^_SNP_ estimate with 95% confidence intervals are shown. Bottom forest plot shows Z-statistic (LDSC h^2^_SNP_ /SE). Red vertical dashed line indicates cut-off where only GWAS with Z- statistic >2 were included in post-GWAS analysis. Note that h^2^_SNP_ estimates reported in the ILAE Consortium on Complex Epilepsies 2023 GWAS manuscript were derived using LDAK resulting in overall higher h^2^_SNP_ estimates than those reported here.

**Supplementary Figure 2: Proportion of shared variants between genetic generalised epilepsy (GGE) and sleep traits, estimated by MiXeR.** Venn diagrams show the overlap between GGE-influencing variants (blue circles) and variants associated with each sleep trait (orange circles). The overlapping region indicates the number of variants shared between GGE and each sleep trait, and the proportion of these shared variants with concordant versus discordant directional effects, as labelled within each panel. Note that GWAS summary statistic for focal epilepsy was underpower for MiXeR, hence not reported.

**Supplementary Figure 3:** UCSC visualisation of genes locate in 1p36.23 (chr1:7711795-8580987; hg 19 reference).

**References**

1.	Watanabe, K., et al., *Functional mapping and annotation of genetic associations with FUMA.* Nature Communications, 2017. **8**(1): p. 1826.

2.	Myers, T.A., S.J. Chanock, and M.J. Machiela, *LDlinkR: An R Package for Rapidly Calculating Linkage Disequilibrium Statistics in Diverse Populations.* Frontiers in Genetics, 2020. **11**.

3.	Wallace, C., *A more accurate method for colocalisation analysis allowing for multiple causal variants.* PLOS Genetics, 2021. **17**(9): p. e1009440.
